# IgE autoantibodies and their association with the disease activity and phenotype in bullous pemphigoid: a systematic review

**DOI:** 10.1007/s00403-017-1789-1

**Published:** 2017-10-25

**Authors:** Ariadne Hadjikyriacou Saniklidou, Patrick J. Tighe, Lucy C. Fairclough, Ian Todd

**Affiliations:** School of Life Sciences, University of Nottingham, Life Sciences Building, University Park, Nottingham, NG7 2RD UK

**Keywords:** Bullous pemphigoid, BP, Immunoglobulin E, IgE, Autoantibodies, Disease severity, Disease activity, Disease course, Clinical phenotype, Clinical manifestations

## Abstract

Bullous pemphigoid (BP) is the most common autoimmune skin disease of blistering character. The underlying pathophysiological mechanism involves an immune attack, usually by IgG class autoantibodies, on the autoantigen BP 180/BPAg2, which is a type XVII collagen (COL17) protein acting as the adhesion molecule between the epidermis and the basement membrane of the dermis. About 40 years ago, following consistent findings of elevated total serum IgE levels in BP patients, it was hypothesized that IgE may be involved in the pathophysiology of BP. Our objective was to determine whether there is strong evidence for an association between IgE class autoantibodies and the clinical severity or phenotype of BP. Three databases were searched for relevant studies and appropriate exclusion and inclusion criteria were applied. Data was extracted and assessed in relation to the study questions concerning the clinical significance of IgE autoantibodies in BP. Nine studies found that anti-BP180 autoantibodies of IgE class are associated with increased severity of BP, whereas two studies did not find such an association. The number of studies which found an association between higher IgE autoantibody levels and the erythematous urticarial phenotype of BP (5) was equal in number to the studies which found no such association (5). In conclusion, higher serum IgE autoantibody levels are associated with more severe clinical manifestations of BP. There is insufficient evidence to support higher IgE autoantibody levels being associated with specific clinical phenotypes of BP.

## Introduction

IgE has traditionally been linked to allergic Type 1 hypersensitivity reactions. However, just over four decades ago, antinuclear autoantibodies of IgE class were detected in RA and SLE patients and it was hypothesized that IgE may have a pathophysiological role in autoimmunity [45]. This association between IgE and autoimmunity was a revolutionary concept in the field of hypersensitivity.

A pathophysiological role of IgE is observed in several autoimmune diseases ranging from systemic ones, like SLE [16], to tissue-specific ones, like Graves’ disease [50]. Furthermore, IgE autoantibodies also seem to be pathogenic in certain allergic conditions, like atopic dermatitis (AD). It is believed that IgE autoantibodies may attack keratinocytes in AD [1], an observation which raises questions as to whether AD is primarily atopic or autoimmune in nature, or a combination of both.

Pemphigoid is a group of autoimmune disorders in which autoantibodies attack the structural proteins comprising the junction of the epidermal and dermal layers of the skin [52]. Pemphigoid disorders manifest themselves clinically through severe blistering of the skin and superficial layers of the mucosa [52]. BP is the most common pemphigoid disorder, accounting for ~ 80% of all cases [49], and is the most common skin autoimmune disease of blistering character. It affects males and females equally, and it is considered mainly a disorder of old age, with the mean age of disease onset being 80. In the UK, it is estimated that there are 43 cases of BP per 1 million of the population [58], with a doubling of reported cases across Europe in the past decade [52]. BP causes relapses of inflammation in the sub-epidermal skin layers, resulting in local or widespread blisters, rash and pruritus. The underlying pathophysiological mechanisms involve an immune attack on the autoantigen BP 180/BPAg2, which is a type XVII collagen (COL17) protein acting as the adhesion molecule between the epidermis and the basement membrane of the dermis. What distinguishes BP from other autoimmune blistering skin diseases is the presence of accumulated IgG on the superficial surface (rather than the dermal surface) of the epidermis in the blistered areas of the skin. Treatment is heavily dependent on the use of topical and systemic corticosteroids.

Studies on BP patients show elevated IgE class autoantibodies with a pathophysiological role. Evidence lies within the detection of IgE autoantibodies targeting the BP180 adhesion molecule and leading to the manifested symptoms [18]. In addition, omalizumab, a humanized monoclonal anti-IgE antibody that was originally designed to control the symptoms of persisting severe asthma following corticosteroid and β_2_ agonist treatment [9], has proven to be effective in treating the symptoms of BP in human subjects [20, 21].

With this information in hand, we decided to carry out a systematic literature review to determine, through analysis of the studies available, whether there is validity in the findings of IgE autoantibodies in BP, and if so, whether the presence of elevated IgE autoantibody levels affects the severity of the phenotype and/or the course of the disease.

## Methods

**Fig. 1:**
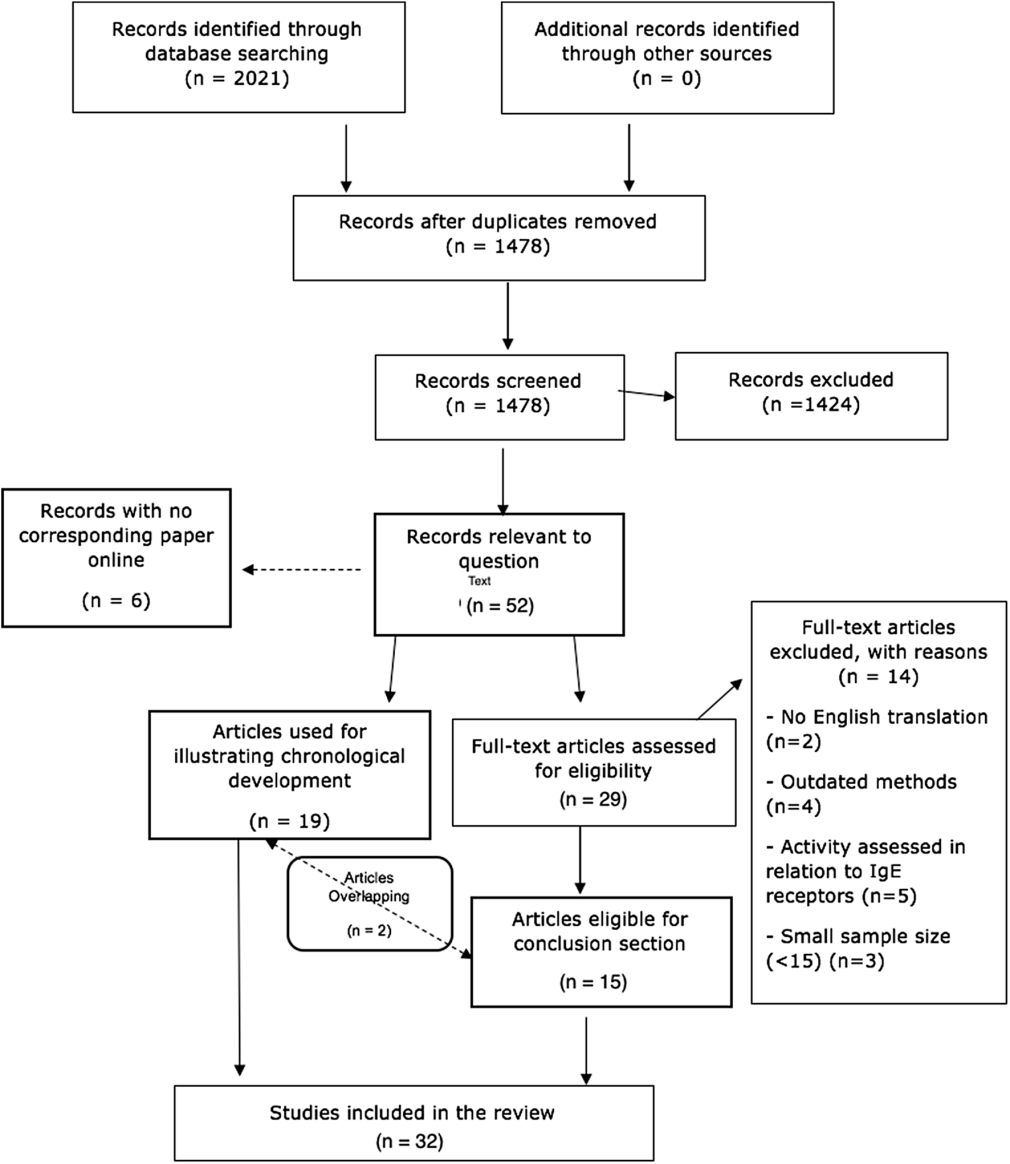
PRISMA 2009 flow diagram used to illustrate the study selection process for the systematic review. This shows the process of searching for records, removing duplicates, screening titles and abstracts, assessing full texts for eligibility and selecting which studies would be used in the analysis

**Fig. 2:**
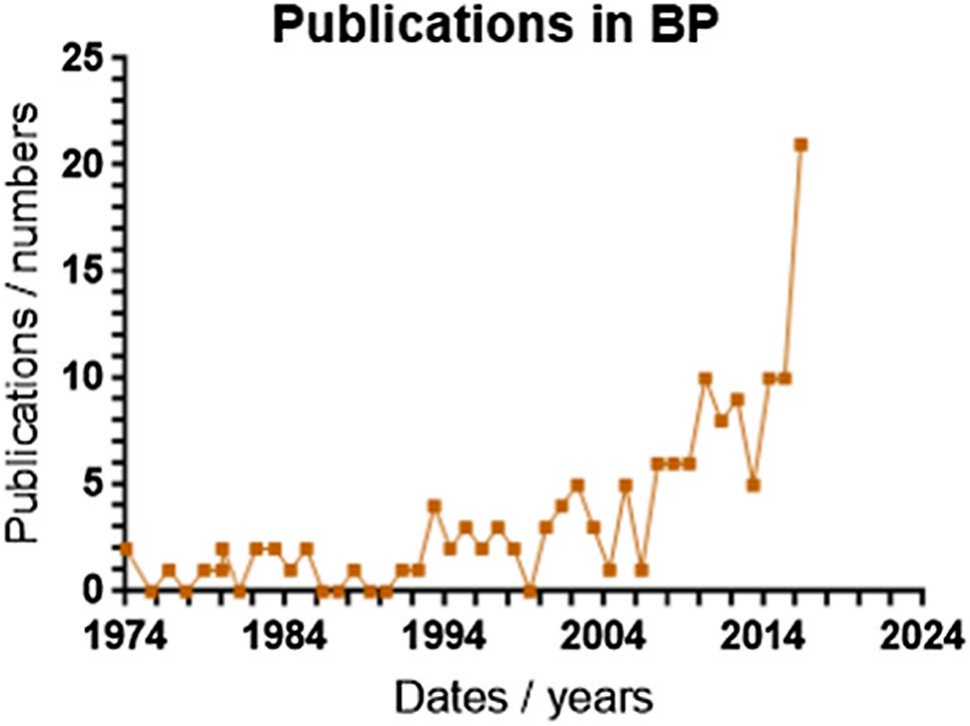
The number of studies published each year between 1974 and 2016 relevant to the relationship between IgE autoantibodies and BP

MEDLINE was accessed through PubMed, EMBASE through OVID and WEB OF SCIENCE through the Wiley Online Library. Each database was searched with the term ‘(ige or immunoglobulin e) and (bullous pemphigoid or bp)’ on 15 October 2016. This yielded a total of 1084 records as follows: MEDLINE, 496; EMBASE, 31; WEB OF SCIENCE, 557. At this initial stage, for comparison between disease, searches were also performed for IgE in chronic idiopathic urticaria (656 records) and IgE autoantibodies in atopic dermatitis (281 records), giving a total of 2021 records. Figure 1 shows the PRISMA 2009 flow diagram used to illustrate the study selection process for the systematic review. This shows the process of searching for records, removing duplicates, screening titles and abstracts, assessing full texts for eligibility and selecting which studies would be used in the analysis. This yielded 33 studies of which 19 were used to document the chronological development of the occurrence of IgE autoantibodies in BP, and 16 provided information to assess the contribution of IgE autoantibodies to the severity and/or clinical phenotype of BP (2 studies provided information relevant to both parts of the review). As illustrated in Fig. 2, of all papers published on the topic in the past 42 years, 50% were published in the last 6 years (2010–2016). This indicates a rapid expansion of interest and investigation in the topic in recent years.

## Results and discussion

### Chronological development in the field of the relationship of IgE and BP

#### IgE and the pathophysiology of BP

The first findings that led to the concept that IgE may be linked to BP were published in 1974. Following this, in the 1970s and 1980s, a variety of studies were conducted to address this hypothesis. During these two decades, blood sampling and testing, immunofluorescence on lesioned skin biopsies and chromatography using dermal infiltrate were the main laboratory techniques employed in this research. As is clearly illustrated below, the role of IgE in BP during this time was only demonstrated in a non-specific fashion: for example, through findings of IgE deposition in skin basement membrane (BM), elevated serum IgE levels and peripheral blood eosinophilia. Inevitably, it was unclear whether the apparent involvement of IgE in BP was of a pathogenic nature, or whether it was simply an epiphenomenon of the disease itself.

In 1974, elevated IgE levels were reported in 70% of patients with bullous pemphigoid [3]. In the same year, a study using direct and indirect immunofluorescence detected moderate staining of IgE on the skin BM of four (out of 16) BP patients, one of whom also had greatly elevated serum IgE [47]. Two years later, a study of tissue eosinophilia in BP lesions of four patients showed eosinophil chemotactic activity in the infiltrate [5]; furthermore, the serum of half of the subjects tested positive for IgE autoantibodies to skin BM. In 1980, a similar study reported that 11 of 25 BP patients had elevated serum IgE levels, and 15 of them tested positive for serum IgE anti-BM autoantibodies by indirect immunofluorescence [44]. A study in 1983 on 28 BP patients found 50% to have peripheral blood eosinophilia; the authors proposed that BP should be considered in the differential diagnosis of skin bullous conditions with peripheral blood eosinophilia [8].

In the early 1990s, the application of newer techniques, such as ELISA, enabled better definition of the relationship between IgE and BP, as it was then possible to identify specific receptors for IgE that were possibly involved in the pathophysiology. By the mid-1990s, it was established that BP involved impaired B cell function, and the soluble form of CD23 (sCD23), a low affinity IgE receptor (FcεRII) on haematopoietic cells, was known to be involved in B lymphocyte growth and differentiation. As a result, the role of CD23 in the pathophysiology of BP was investigated and elevated levels of sCD23 were detected in the sera of BP patients [24]. Furthermore, there was significant correlation between sCD23 and serum IgE levels in the BP patients; this correlation was not apparent in the sera of non-BP control subjects. The authors concluded that sCD23 was an important index for monitoring the disease in relation to IgE abnormalities and impaired B lymphocyte function. A year later, the understanding of the involvement of sCD23 in BP was further advanced when it was reported that levels of sCD23 in the dermal bullae infiltrates of ten BP patients were significantly higher than in the infiltrates of suction bullae formed in ten non-BP control subjects [51]. Again, there was correlation between the serum levels of sCD23 and IgE levels in all ten BP patients.

Some pivotal studies in the development of the field showed specific autoantigens in the hemidesmosomal proteins of the skin to be the targets of autoreactive IgE antibodies. As discussed below, BP180 and BP230 are major autoantigens targeted by IgE autoantibodies in BP. BP180 (BPAg2), a transmembrane protein, was the first autoantigen characterized in BP and was found to have collagenous repeats in its ectodomain capable of forming collagen-like helices [27]; indeed, BP180 was later renamed Collagen XVII. BP230 (BPAg1) was first identified using sera from BP patients and was found to be located on the intracellular domain of the hemidesmosome where it functions as a plakin family protein, associating keratin filaments to the hemidesmosome [53]. As a consequence of identifying these two proteins as targets of IgE autoantibodies, by the mid-1990s, the role of IgE in BP was more widely accepted as being pathogenic rather than being an epiphenomenon.

A study in 1996 using a radioimmunoassay reported 12 out of 19 BP patients to have IgE autoantibodies against the BP55 antigen [15]. Two years later, a study examined the antigenic specificity of IgE autoantibodies in 39 BP serum samples using ELISA, immunoblotting and immunofluorescence microscopy: 18 of the samples contained IgE autoantibodies against either the BM or a 230 kDa epidermal antigen (BP230) [25]. The epitopes recognized by the autoantibodies mapped primarily to the C-terminal end of the protein and there was strong correlation between circulating IgE autoantibodies and serum IgE levels. However, this study found no sera containing anti-BP180 IgE autoantibodies. In 2000, immunoblotting and ELISA were used to show that IgE and IgG4 isotypes preferentially react with two epitopes within the ectodomain (NC16A) of BP180 [18]. Another study used double-labelling immunofluorescence to identify a potential effector function of IgE autoantibodies associated with their reactivity to BP180 auto-antigen: of the 30 BP patients studied, 70% of untreated patients had elevated total IgE levels and 86% had detectable anti-BP180 IgE in their serum [17]. IgE-coated mast cells were detected in perilesional skin, which were also coated with BP180 peptides. BP180-stimulated histamine release was notably higher in basophils obtained from BP patients who received no treatment in contrast to patients who either received treatment for BP or the healthy control subjects. The attack on BP180 by autoreactive IgE continued to be the main focus of the investigations for the rest of the decade. Fine mapping of the antigenic sites of NC16A ectodomain of BP180 showed similar reactivity patterns for IgE and IgG, with subregion-2 being the major site recognized by both [23]. However, antigen-specific histamine release by basophils occurred only in the presence of IgE autoantibodies specific for NC16A. In 2009, a study using immunoblotting found that 16 of 18 serum samples contained autoreactive IgE specific for an epitope of the intracellular domain (ICD) of BP180, suggesting that the ectodomain NC16A is not the only target region of IgE on BP180 [19]. The target sites on the ICD of BP180 play an important role in the incorporation of proteins into hemidesmosomal structures. The authors suggested that this could be a possible pathogenic mechanism of IgE in BP, since tampering with the interaction of BP180 with other hemidesmosomal constituents could lead to the dermal–epidermal separation observed in the affected skin of BP patients.

An interesting recent observation concerning the pathophysiology of IgE in BP indicates that IgE may have FcR-independent effects: IgE was found to stimulate FcR-independent release of IL-6 and IL-8 by keratinocytes in vitro [41]. When the experiment was repeated using an organ (skin) culture, IL-6 and IL-8 were similarly released. Cytokine release was also accompanied by a decrease in the number of hemidesmosomal proteins located in the BM zone.

#### IgE and the diagnosis of BP

A highly sensitive method for detecting anti-BP180 NC16A IgE autoantibodies in sera of BP patients was reported in 2009 [39]. The assay detected IgE autoantibodies in 77% of sera tested, a frequency significantly higher than previously reported and equivalent to that of anti-BP180 NC16A IgG autoantibodies. The results of the study ranked the ELISA method as the most sensitive, when compared with other techniques such as immunoblotting and IIF, and that testing for both IgG and IgE anti-BP180 NC16A may be a more efficient indicator of disease severity and predictor of treatment effectiveness. Nevertheless, the authors stressed that NC16A is not the only target of anti-BP180 autoantibodies, and that BP180 is not the only autoantigenic target in BP. Thus, an NC16A-specific ELISA alone may underestimate the actual reactivity of autoantibodies with BP180 and other autoantigens in BP.

More recently, Pomponi et al. evaluated the efficiency of the ISAC® microarray system in detecting IgE and IgG antibodies in BP patients [46]. The experimental version of ISAC® that was used included BP180 NC16A and was able to replicate the results of ELISA. These improvements in the diagnostic techniques for the detection of autoantibodies involved in BP are of great importance in reducing discrepancies that arise in the literature due to the heterogeneity in the sensitivity of various laboratory assays.

#### IgE and the treatment of BP

The hypothesis of IgE having a pathogenic role in BP has been further established through the successful treatment of BP patients with omalizumab, a humanized monoclonal anti-IgE antibody which binds to IgE [60]. In 2009, Fairley et al. reported the use of omalizumab to treat a BP patient who was unresponsive to steroid treatment [21]. The team justified the decision based on the study of Dimson et al. [17] which reported that 70% of untreated BP patients presented with elevated total serum IgE levels and 86% presented with anti-BP180 NC16A IgE autoantibodies. Clinical improvement was observed within 16 weeks of commencing omalizumab treatment. By week 1 of treatment, clinical improvements included a decrease in pruritus and in bullous count by 44%. By week 16, there was a 45% decline in the skin involved in urticarial lesions and a drop in peripheral blood eosinophil count from 3427 to 887/mm^3^ (normal ≤ 475/mm^3^). Four months after discontinuation of treatment, the patient reported pruritus and new bullae formation, but re-initiation of omalizumb resulted in resolution of bullae and subsidence of pruritus. This was the first treatment targeting IgE in a BP patient and it was also the first in vivo demonstration of the pathogenicity of IgE class autoantibodies in BP. Similarly, in 2012, Dufour et al. [20] reported the treatment with omalizumab of a 5-month-old boy suffering from infant BP (IBP), who was unresponsive to steroids. Omalizumab treatment resulted in a decline in the number of bullae and urticarial lesions and overall disease control was achieved by day 25 of treatment. Omalizumab was continued for the next 7 months and no clinical relapse occurred during this period. This observation supported the efficacy of omalizumab in treating IBP. Following these reports of single patient cases, a study was undertaken in which six patients were treated with omalizumab and monitored for up to 42 months [60]. Five of them benefited therapeutically: there was inhibition of new bullae formation, subsidence of pruritus, a decline in eosinophil count and a decreased need for immunosuppressant medication. Despite this being an uncontrolled study, the authors felt that the results were sufficiently clear-cut to conclude that omalizumab can neutralize IgE reactivity in patients with BP and control the disease.

### IgE in relation to disease activity and clinical phenotype of BP

As the role of IgE in the pathophysiology of BP became better understood as a pathogenic one, hypotheses about how its presence in BP may affect the disease severity/activity and/or clinical manifestations (i.e. phenotype) were put forward. Of the earliest papers to explore a possible correlation between IgE and BP activity was that of Asbrink and Hovmark [4], which suggested that total serum IgE was a better measure of disease activity in contrast to IIF. Since then, several studies conducted in the field have addressed how total serum IgE or, more commonly, the levels of IgE autoantibodies in the serum, can potentially affect the disease severity/activity and/or the phenotype of BP. This review has assessed the outcomes of 16 primary studies (details summarized in Table 1a, b) conducted between 2000 and 2016, and one Letter to the Editor, which address this issue.

**Table 1:** Summary of the data extracted from the primary full articles used in assessing the association of IgE with the severity and clinical phenotype of BP

First author [Ref]	Year	Journal of publication	Type of article	Study design	Setting details	Sample size	Patient demographics	Description of BP diagnosis criteria present	Comorbidities
a									
Döpp et al. [18]	2000	J Am Acad Dermatol	Journal article	Cohort	Single centre; University of Würzburg	18	11 males and 7 females; range of age 50–91 years and a median of 72	No	Not discussed
Cozzani et al. [14]	2001	J Eur Acad Dermatol Venereol	Journal article	Cohort	Multi-centre; The Dermatologic Clinic of the University of Genoa and the Division of Dermatology of S. Martino, Galliera, Sampierdarena, Imperia, Savona and La Spezia Hospitals	32	13 males and 19 females; range of age 45–92 years and a mean age of 74	Yes	Discussed
Kelly et al. [35]	2007	J Allergy Clin Immunol	Meeting Abstract	Not clear	Single-centre; University of Utah	140 and 48	No information	No	Not discussed
Ishiura et al. [31]	2008	J Dermatol Sci	Journal Article	Case–Control	Multi-centre; University of Tokyo Hospital and Kanazawa University Hospital	67	35 males and 32 females; range of age 17–93 years and mean of 72.4	Yes	No comorbidities that could affect serum IgE or eosinophil levels
Iwata et al. [32]	2008	Arch Dermatol	Jounral Article	Retrospective case series analysis	Single-centre; Nagasaki University Graduate School of Biomedical Science, Department of Dermatology	37	18 males and 19 females; mean age of 75	Yes	Not discussed
Messingham et al. [39]	2009	J Immunol Methods	Journal Article	Cohort	Single-centre; University of Iowa	43	No information	Yes	Not discussed
Yayli et al. [59]	2011	Br J Dermatol	Journal Article	Retrospective study	None provided	44	23 males and 21 females; range of age 46–94 years and a mean age of 76.5	Yes	Not discussed
Messingham et al. [40]	2014	PLoS One	Journal Article	Case–control study	Single-centre; University of Iowa	48	25 males and 23 females; range of age 59–97 years and a mean of 78.2	Yes	Not discussed
Moriuchi et al. [42]	2015	J Dermatol Sci	Journal Article	Retrospective study	Single-centre; Hokkaido University Graduate School of Medicine, Department of Dermatology	100	47 males and 53 females; range of age 41–99 years and a mean age of 72.2	Yes	Not discussed
Ma et al. [38]	2015	J Dermatol Sci	Letter to Editor	Case–control	Single-centre; University Hospital in China (name not mentioned)	41	19 males and 22 females; mean age of 69.37	Yes	Not discussed
Bing et al. [7]	2015	Arch Dermatol Res	Journal Article	Case–control	Single-centre; Hospital in China (name not mentioned)	37	21 males and 16 females; range of age 29–93 and a mean of 69.08	Yes	Not discussed
Kalowska et al. [34]	2016	Acta Derm Venereol	Journal Article	Retrospective study	Medical University of Warsaw, Department of Dermatology	77	22 males and 55 females; range of age 56–97 years with a mean of 78.6	Yes	Not discussed
Cho et al. [11]	2016	J Dermatol Sci	Letter to Editor	Retrospective study	Single-centre; National Taiwan University Hospital	17	9 males and 8 females; range of age 11–77 years and a mean age of 72	Yes	Not discussed
Hashimoto et al. [26]	2016	Br J Dermatol	Journal Article	Retrospective study	Single-centre; Kurume University	36	8 males and 28 females; range of age 1–90 years and a mean age of 63.6	Yes	Not discussed
van Beek et al. [57]	2016	JAMA Dermatol	Journal Article	Cohort study	Single-centre; University clinic of Lübeck	153 (Cohort 1: 65, Cohort 2: 52, Cohort 3: 36)	Cohort 1 (underwent ELISA for IgE BP180 NC16A aabs): 25 males and 40 females, mean age of 74.6 years; Cohort 2 (underwent BPDAI clinical evaluation): 23 males and 29 females and a mean age of 78.2 years; Cohort 3 (negative for anti-BP180 NC16A IgG and underwent evaluation of the diagnostic importance of serum anti-BP180 IgE): 22 males and 14 females and a mean age of 74.5 years	Yes	Not discussed

First author [Ref]	Year	Variable X; IgE	Method of measurement	Variable Y	Method of measurement	Intervention/s	Duration	Control/comparison	Outcome
b									
Döpp et al. [18]	2000	Anti-BP180 NC16A aabs	Immunoblotting and ELISA	Disease Activity	No method given	Oral prednisolone + dapsone or doxycycline + nicotinamide	8 weeks	50 healthy controls; no matching mentioned	Serum concentrations of anti-BP180 IgE aabs parallel disease activity
Cozzani et al. [14]	2001	Total serum IgE	IIF	Disease activity and phenotype	Serum IgE via IIF; clinical symptoms	Treatment for BP; no drug mentioned	6 months	No mention	Total serum IgE does not correlate with disease activity; more correlation with bullous and not urticarial phenotype
Kelly et al. [35]	2007	Total serum IgE	ELISA	Disease activity	No method given	Non-interventional study	–	No mention	Serum IgE highly correlated with disease activity; serum IgE levels increased as BP180 and BP230 ELISA levels increased
Ishiura et al. [31]	2008	anti-BP180/230 aabs	ELISA	Disease activity	% of skin covered with lesions (disease severity), disease duration, serum IgE levels, serum and local eosinophil count	Non-interventional study	–	36 healthy controls; 18 males and 18 females; range of age 51–85 years and a mean of 70.1; age and sex matched	Affected areas negatively correlated with anti-BP230 IgE aabs
Iwata et al. [32]	2008	anti-BP180/230 aabs + total serum IgE	ELISA	Disease activity	Scale of 1 (remission)—4 (highest activity) au	Non-interventional study	–	26 healthy controls; age and sex matched; 6 pemphigus vulgaris and 5 pemphigus foliaceus disease comparison patients	Anti-BP180 IgE aabs associated with broader skin lesions, % of skin with lesions, required steroid dosage, longer duration of treatment necessary for remission and more intensive therapies (immunosuppressive agents). Thus, anti-BP180 IgE aabs parallels disease activity and causes a severe form of BP (anti-BP230 IgE do not correlate with disease activity)
Messingham et al. [39]	2009	Anti-BP180 NC16A aabs	ELISA + western blot	Disease activity	Scale of 1–4 (from least to most severe)	Treatment for BP; no drug mentioned	–	55 healthy controls; age and sex matched; 6 disease comparisons with epidermolysis bullosa acquisita (EBA) and 21 disease comparisons with SLE	Anti-BP180 NC16A IgE aabs decrease over the treatment course with improvement in disease severity and vice versa
Yayli et al. [59]	2011	IgE deposits in biopsy specimen	DIF microscopy	Disease phenotype	Presence of specific clinical symptoms	Non-interventional study	–	13 MMP disease comparison patients; 4 males and 9 females; range of age 44–84 years and a mean age of 68.7	5/18 of patients with linear (unspecified) IgE deposits along the BM zone had urticarial papules and flakes and increased pruritus
Messingham et al. [40]	2014	anti-BP180 NC16A aabs + total serum IgE	ELISA	Disease severity	BP index scaled from 1 to 6 (remission to severe disease) and/or the BPDAI criteria scaled from 0 to 120 (least to most severe)	Non-interventional study	–	58 healthy control and 25 disease comparisons (dermatology patients with other autoimmune diagnoses); age and sex matched	Correlation between anti-BP180/230 aabs and disease activity BPDAI
Moriuchi et al. [42]	2015	IgE aabs at BM zone	ELISA and IIF	Disease severity and phenotype	Presence of specific clinical symptoms	Non-interventional study	–	None mentioned	No correlation between anti-BP180/230 IgE aabs and disease severity; predominant IgE deposition to BM zone alters the pattern of clinical manifestations of BP, e.g. development of erythrodermic BP or pemphigoid nodularis (a rare form of BP)
Ma et al. [38]	2015	anti-BP180 aabs	ELISA	Disease severity	% area of skin lesions, dosage of prednisone and duration of treatment for remission	Non-interventional study	–	30 healthy controls and 16 disease comparisons with pemphigus vulgaris	No positive correlation between IgE anti-BP180 aabs with disease severity; however, correlated slightly with broader areas of skin lesions larger prednisone dosage and a longer duration of treatment for a more effective control
Bing et al. [7]	2015	Anti-BP180 NC16A in serum and dermal infiltrate	ELISA	Disease activity	Scale of 1–5 (least to most skin eruptions/blisters) au; one extra point for mucosal involvement	Non-interventional study	–	28 healthy controls; 13 males and 15 females; range of age 60–82 years and a mean of 67.32; 18 patients with pemphigus vulgaris and 1 with Stevens–Johnson syndrome as disease comparisons; 12 males and 7 females, range of age 33–86 years and a mean age of 54.05	Anti-BP180 NC16A IgE levels reflected disease severity generally; however, Spearman test showed −ve *r* between disease activity scores in the initial 1–2 months (early stage of BP) and anti-BP180 NC16A IgE aab titres
Kalowska et al. [34]	2016	Anti-BP180 NC16A aabs + total serum IgE	ELISA + chemiluminescence	Disease activity	% of body surface area affected	Non-interventional study	–	29 healthy controls with no parasitic infections, autoimmune diseases or allergic reactions; 21 males and 8 females; range of age 62–87 years and a mean of 70.5	+ve Pearson *r* test between anti-BP180 NC16A IgE aabs + total serum IgE and disease severity in the active stage of BP; significant correlation between clinical remission of BP and serum IgE
Cho et al. [11]	2016	Anti-BP180/230 aabs	ELISA	Disease severity	BPDAI scale	Non-interventional study	–	None mentioned	High serum anti-BP180 IgE aabs correlated with high BPDAI scores; also correlation with urticaria/erythema scores, but not eruptions/blister scores
Hashimoto et al. [26]	2016	IgE anti-BP180/230 aabs	ELISA	Disease severity and phenotype	Developed their own criteria of disease severity and phenotype; disease severity score of 1–2, BP phenotypes included bullous, erythematous, bullous/erythematous, nodular	Non-interventional study	–	None mentioned	ELISA values of anti-BP180 IgE aabs correlated with severity scores 1 and 2, but there was no statistical significance of such correlation regarding anti-BP230 IgE aabs; no significant association between BP180/230 and the bullous/erythematous or erythematous phenotypes, but statistically significant association with nodular phenotype
van Beek et al. [57]	2016	Anti-BP180 NC16A	ELISA	Disease activity and phenotype	BPDAI score for severity; clinical presentation determined by the total BPDAI score (0-200 points) [= BPDAI blister/erosion + BPDAI urticaria/erythema]	Non-interventional study	2008–2014 (6 years)	30 disease comparisons with pemphigus vulgaris or pemphigus foliaceus (14 males and 16 females with a mean age of 54.4 years); 49 healthy controls with non- inflammatory dermatoses (21 males and 28 females with a mean age of 81.6 years) and 127 healthy controls undergoing allergy testing for IgE levels	47/117 patients tested positive for anti-BP180 NC16A serum IgE aabs and there was correlation with their disease activity (BPDAI score); no correlation between BP180 NC16A IgE aabs with urticarial or erythematous lesions or the classic phenotype of blisters and eruptions; total serum IgE correlated with the BPDAI score of pruritus

#### IgE and the disease severity/activity of BP

Disease severity of BP has been defined in a variety of ways over the years. The severity of BP used to be assessed by counting the number of bullae on the skin of the patient. Joly et al. and Bernard et al. [6, 33] reported the use of this method: the larger the number of bullae, the more points were allocated for disease severity. Tsuji-Abe et al. and Roujeau et al. [48, 55] reported the use of a similar method, but instead of counting individual bullae, it was the percentage of skin affected by lesions that was taken into consideration. Ishiura et al. [31], Iwata et al. [32] and Messingham et al. [39] also reported the use of this method and the development of scales (unofficial, self-defined scoring scales) for quantifying disease severity. In 2012, the bullous pemphigoid disease area index (BPDAI) was introduced. This is an objective scale which continues to be used for assessing the % of skin affected by BP-related lesions (both bullous and erythematous) and degree of pruritus [34]. The scale ranges from 0 to 120 and the total BPDAI score can be calculated by adding together the scores for bullae, erythematous lesions and pruritus [57].

As shown in Table 2, nine studies [7, 11, 18, 26, 32, 34, 39, 40, 57] found that anti-BP180 autoantibodies of IgE class are associated with increased severity of BP, in contrast to two studies [38, 42] which did not find such an association. The two studies which reported no association did not seem to have any major differences in conduct from the rest of the studies which did report an association. No studies reported an association between anti-BP230 IgE autoantibodies and disease severity, while four studies [26, 31, 32, 42] stated that anti-BP230 IgE autoantibody serum levels or deposition in the skin BM showed no correlation with disease severity.

**Table 2:** The number of studies indicating that a specific parameter of IgE can or cannot affect the disease severity/activity of BP [Refs]

	Direct correlation with anti-BP180 IgE aabs	No correlation with anti-BP180 IgE aabs	Direct correlation with anti-BP230 IgE aabs	No correlation with anti-BP230 IgE aabs	Direct correlation with total serum IgE	No correlation with total serum IgE
Disease severity/activity	9 [7, 11, 18, 26, 32, 34, 39, 40, 57]	2 [38, 42]	0	4 [26, 31, 32, 42]	3 [11, 34, 35]	1 [14]

Since it has been demonstrated that total serum IgE levels correlate directly with the levels of IgE specific for BP180 and BP230 antigens [35] and that there is a good positive correlation between circulating IgE autoantibodies and total serum IgE [25], some studies have sought to determine whether disease severity correlates with total serum IgE levels: three studies reported a positive correlation between total serum IgE levels [11, 34, 35] and disease severity and one study [14] reported no correlation.

Overall, the high proportion of studies reporting an association between anti-BP180 IgE autoantibodies and severity of BP compared to the studies reporting no such association (9:2) favours a correlation between the presence of IgE autoantibodies and increased disease severity.

#### IgE and the clinical presentation of BP

Bullous pemphigoid does not manifest as a single phenotype. The typical bullous phenotype involves local or widespread blisters and eruptions; however, these can be preceded by lesions of an urticarial nature on erythematous skin. One of the less common forms of BP is pemphigoid nodularis (nodular phenotype) which is characterized by highly pruritic lesions, often formed on nodular skin [12].

Table 3 shows the numbers of studies that have reported correlation (or lack of correlation) of various IgE parameters to the different phenotypes of BP [11, 14, 26, 42, 57, 59]. Overall, these studies indicate that IgE autoantibodies are not specifically associated with the blisters and eruptions seen in the typical presentation of BP. The urticarial presentation of the condition has been the most investigated phenotype of BP with respect to parameters of IgE expression. However, the number of reports that IgE (total or autoantibody) levels are associated with urticarial lesions is equal to the number that found no such associations. There is thus insufficient evidence at present to conclude from studies in patients that IgE autoantibodies are associated with specific clinical phenotypes of BP.

**Table 3:** The number of studies reporting that a specific parameter of IgE can or cannot affect the clinical manifestation (phenotype) of BP [Refs]

	Correlation with anti-BP180 IgE aabs	No correlation with anti-BP180 IgE aabs	Correlation with anti-BP230 IgE aabs	No correlation with anti-BP230 IgE aabs	Correlation with unspecified IgE aabs	Correlation with total serum IgE	No correlation with total serum IgE
Bullous	0	2 [11, 57]	0	0	0	1 [14]	0
Urticarial Erythematous	2 [11, 42]	3 [7, 26, 57]	1 [42]	1 [26]	1 [59]	0	1 [14]
Bullous/erythematous	0	1 [26]	0	1 [26]	0	0	0
Nodular	2 [26, 42]	0	2 [26, 42]	0	0	0	0

Amber [2] suggested that two possible problems in establishing an association between IgE autoantibodies and the urticarial stage of BP could be variable sensitivity in detecting IgE autoantibodies in BP patients and insufficient discrimination of distinct clinical phenotypes within the spectrum of the disease (see also reply by Zuo [63]). Two other lines of investigation that indirectly provide evidence relevant to this issue are studies of BP in murine models, and studies of IgE autoantibodies in other skin diseases:

Firstly, whereas IgG-mediated murine models lack urticarial erythema and eosinophil infiltration, these features occur in human skin engrafted on to SCID mice inoculated with monoclonal IgE specific to the BP180 ectodomain [62]. A similar model has been developed using human skin-engrafted nude mice inoculated with IgE from BP patients [22]. Direct immunization of mice with fragments of BP180 also induces skin lesions associated with raised IgE and infiltration of eosinophils [28].

Secondly, autoantibodies of IgE class have been reported in patients with other inflammatory skin diseases. IgE autoantibodies specific for double-stranded DNA [10] and for thyroid autoantigens [10, 13] have been described in some patients with chronic idiopathic urticaria; these autoantibodies may contribute to disease symptoms by activating mast cells and basophils [37]. IgE autoantibodies specific for a variety of autoantigens have been reported in some atopic dermatitis patients [29, 30, 43, 54, 56, 61] and appear to correlate with disease severity and chronicity [36, 54].

### Limitations of this systematic review

As shown in Table 1, the studies used in this review were subject to several limitations. Most were conducted in a single-centre fashion, and only a few countries (notably, the USA, Japan, China and Germany) have been conducting the majority of research in this area. As a result, it is not known whether the conclusions of these studies are generalizable to all ethnicities. In addition, most of the studies used sample sizes that are small—this problem may arise because of the relative rarity of BP. In general, little information has been given about the subjects involved, other than sex and age; for example, about their overall health and any comorbidities they have that could constitute confounding factors, or about the methods whereby they were recruited. Lack of such information could be a warning for selection, exclusion and sampling bias. Another potential problem is the ambiguity in the full spectrum of signs, symptoms and phenotypes of BP, leading to a vague consensus on the manifestations of the disease. This poses a risk of classification bias when selecting patients for a study. Moreover, procedural errors could lead to bias as well. For instance, several studies have reported an uncertain degree of sensitivity of the methods used in determining IgE autoantibody concentrations, and blinding of the scientists conducting the experiments is not often mentioned, raising possible detection bias. The diversity in the scales used to assess the disease severity of BP contributes further to procedural inaccuracies. The lack of healthy controls and disease comparisons in numerous studies poses a risk of yielding results of questionable reliability. Furthermore, most studies included in this review are retrospective studies, which pose an increased risk of recall bias and the influence of confounding factors compared to prospective studies. As with all systematic reviews, there is inevitably a risk of publication bias as the conclusions of the review are based on published data, which can be a problem since positive outcomes are three times more likely to be published than are negative outcomes. No grey literature was used in this review and some data were unavailable for us to use. Furthermore, there is a high degree of heterogeneity in the studies used, particularly with respect to the sample sizes and characteristics, the criteria for diagnosing BP, the type of variables investigated and the methods used for measuring the variables, the use of control/comparison groups and the actual outcomes.

## Conclusion

This review’s results support the conclusion that the higher the serum IgE autoantibody levels are, the more severe the manifestation of BP will be; however, they do not support the possibility that higher IgE autoantibody levels promote a more urticarial presentation of BP. It is fair to say that there is ambiguity as to whether IgE can be held accountable for the full spectrum of BP manifestations, or whether its effects are more relevant to the initial stages of the disease. Further research is needed in the future to establish more solid conclusions. There is a need for more multi-centre prospective cohort studies which would help to eliminate uncertainties in the field, particularly with respect to a possible association between the occurrence of IgE class autoantibodies and the clinical phenotype of BP.
